# *catena*-Poly[{di-μ-isonicotinato-bis[di­aqua­isonicotinatoeuropium(III)]}-μ-isonicotinato-[diisonicotinatocopper(II)]-μ-isonicotinato]

**DOI:** 10.1107/S1600536808038476

**Published:** 2008-11-22

**Authors:** Miao-Ling Huang

**Affiliations:** a Department of Chemistry and Life Sciences, Quanzhou Normal University, Fujian 362000, People’s Republic of China

## Abstract

The title compound, [CuEu_2_(C_6_H_4_NO_2_)_8_(H_2_O)_4_]_*n*_, displays a one-dimensional chain structure. The four-coordinate Cu^II^ ion (site symmetry 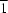) adopts a *trans*-CuN_2_O_2_ geometry and is bridged by two carboxyl­ate groups from two isonicotinate ligands. The Eu^III^ ion adopts a distorted square-anti­prismatic geometry, being coordinated by four O atoms from bridging carboxyl­ate groups of four isonicotinate ligands, two O atoms from chelating carboxyl­ate groups of one isonicotinate ligand and two O atoms from coordinated water mol­ecules; adjacent Eu^III^ ions in the chain are related by inversion. The water mol­ecules interact with the ligands *via* O—H⋯N hydrogen bonds [O⋯O = 2.782 (3)–2.881 (3) Å], which link the chains into a three-dimensional structure.

## Related literature

For background literature, see: Zhao *et al.* (2006 ▶); Ma *et al.* (2001 ▶). For related structures, see: Liang *et al.* (2007 ▶); Zhang *et al.* (2005 ▶); Deng *et al.* (2008 ▶).
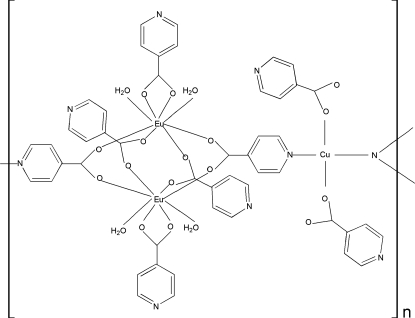


## Experimental

### 

#### Crystal data

[CuEu_2_(C_6_H_4_NO_2_)_8_(H_2_O)_4_]
*M* *_r_* = 1416.34
Monoclinic, 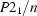

*a* = 9.5218 (9) Å
*b* = 15.0371 (13) Å
*c* = 18.2850 (16) Å
β = 93.822 (1)°
*V* = 2612.2 (4) Å^3^
*Z* = 2
Mo *K*α radiation
μ = 2.86 mm^−1^
*T* = 295 (2) K
0.40 × 0.30 × 0.27 mm

#### Data collection

Bruker SMART CCD area-detector diffractometer
Absorption correction: multi-scan (*SADABS*; Sheldrick, 2003 ▶) *T*_min_ = 0.397, *T*_max_ = 0.517 (expected range = 0.355–0.462)
18387 measured reflections
4853 independent reflections
4222 reflections with *I* > 2σ(*I*)
*R*_int_ = 0.036

#### Refinement

*R*[*F* ^2^ > 2σ(*F* ^2^)] = 0.021
*wR*(*F* ^2^) = 0.049
*S* = 1.04
4853 reflections
359 parameters
6 restraints
H-atom parameters constrained
Δρ_max_ = 0.36 e Å^−3^
Δρ_min_ = −0.37 e Å^−3^

### 

Data collection: *SMART* (Bruker, 2001 ▶); cell refinement: *SAINT* (Bruker, 2003 ▶); data reduction: *SAINT*; program(s) used to solve structure: *SHELXS97* (Sheldrick, 2008 ▶); program(s) used to refine structure: *SHELXL97* (Sheldrick, 2008 ▶); molecular graphics: *ORTEP-3 for Windows* (Farrugia, 1997 ▶); software used to prepare material for publication: *SHELXTL* (Sheldrick, 2008 ▶).

## Supplementary Material

Crystal structure: contains datablocks global, I. DOI: 10.1107/S1600536808038476/pv2118sup1.cif

Structure factors: contains datablocks I. DOI: 10.1107/S1600536808038476/pv2118Isup2.hkl

Additional supplementary materials:  crystallographic information; 3D view; checkCIF report

## Figures and Tables

**Table 1:** Hydrogen-bond geometry (Å, °)

*D*—H⋯*A*	*D*—H	H⋯*A*	*D*⋯*A*	*D*—H⋯*A*
O10—H4*W*⋯O8^i^	0.83	1.97	2.782 (3)	165
O9—H2*W*⋯O1^i^	0.83	1.98	2.790 (3)	164
O10—H3*W*⋯N1^ii^	0.83	2.06	2.881 (3)	169
O9—H1*W*⋯N4^iii^	0.83	2.00	2.807 (3)	161

Symmetry codes: (i) 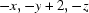; (ii) 
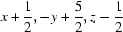
; (iii) 
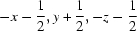
.

**Fig. 1.**
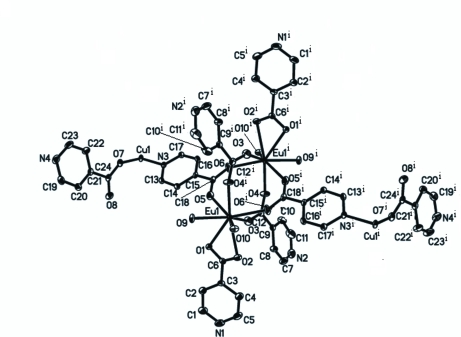
The ORTEP-3 (Farrugia, 1997) drawing of the title compound. Displacement ellipsoids are drawn at 30% probability level. Symmetry codes: (i) -x + 1, -y + 2, -z.

**Fig. 2.**
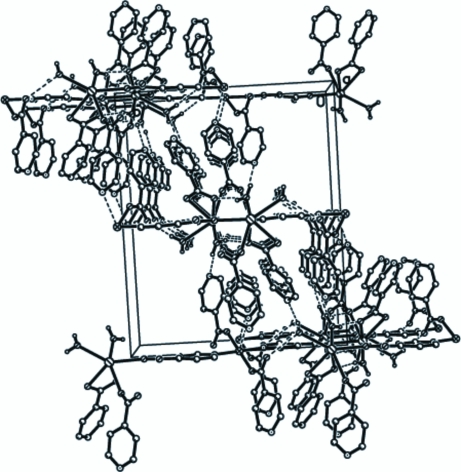
Projection showing the three-dimensional structure formed by H-bonding interaction of the compound (I).

## supplementary crystallographic information

### Comment

In recent years, the study of heterometallic lanthanide-transition metal polymers has played an important role due to their potential applications in catalytic, magnetic and hochtemperatursupraleiter materials (Zhao *et al.*, 2006; Ma *et al.*, 2001). Isonicotinic acid is a good linear bridging ligand with oxygen and nitrogen donors on opposite sides. the crystal structures of heterometallic lanthanide-transition metal complexes with isonicotinate ligand have been reported (Liang *et al.*, 2007; Zhang *et al.*, 2005; Deng  *et al.*, 2008;). In order to extend further the study of these compounds, the title complex, (I), has been synthesized and its structure is presented in this article.

The molecular structure and the crystal packing diagram of the title compound are shown in Figs. 1 and 2, respectively. The complex structure is a one-dimensional chain consisting of two metal centres - Cu(II) and Eu(III), which are linked to each other by tridentate bridging isonicotinate ligands. It is worthwhile to note that one-dimensional chain is an axial-symmetric structure. Each Eu(III) cation is of an eight coordination consisting of eight oxygen atoms from four bridging carboxylate O atoms of four isonicotinate ligands [Eu—O distances ranging from 2.341 (2) to 2.366 (2) Å], two from chelating carboxylate O atoms of one isonicotinate ligand [Eu—O distances 2.477 (2) and 2.5428 (19) Å] and two water molecules [Eu—O distances 2.4133 (19) and 2.4548 (19) Å]. The O—Eu—O bond angles are in the range from 51.88 (6) to 152.34 (8)°. Therefore, the coordination polyhedron can be described as a distorted square antiprism. Two Eu(III) ions are connected by four bridging carboxylate groups of four isonicotinate ligands with a Eu···Eu distance of 4.5265 (4) Å. The Cu(II) cation, lies on an inversion center and is of four-coordination with an quadrilateral planar geometry, in which two coordinated oxygen atoms belong to two monodentate coordinating carboxylate O atoms of two isonicotinate ligands [Cu—O distance 1.9867 (19) Å] and two nitrogen atoms from two tridentate bridging isonicotinate ligands [Cu—N distance 2.011 (2) Å]. The O—Cu—N bond angles lie in a very narrow range of 89.26 (9) to 90.74 (9)°. Two weak Cu—N bonds with a distance of 2.654 (3)Å on both sides of the quadrilateral planar copper center are observed, in which the coordination N atoms come from pyridyl groups of two isonicotinate ligands bridging two Eu(III) ions *via* carboxylates in two adjacent molecular chains. The water molecules are hydrogen bonded to the N atoms of the pyridyl groups of the ligands *via* O—H···N type hydrogen bonds which link the complex into a three-dimensional structure (details of hydrogen bonding geometry are given in Table 1).

### Experimental

A mixture of Eu_2_O_3_ (0.1811 g, 0.5 mmol), CuO (0.0801 g, 1 mmol), isonicotinic acid (0.4923 g, 4.0 mmol), and H_2_O (20.0 ml) was sealed in a 40 ml Teflon-lined stainless steel reactor, heated in an oven at 413 K for 72 h, and then slowly cooled to room temperature. The blue block single crystals suitable for X-ray analysis were collected.

### Refinement

H atoms bonded to C atoms were placed geometrically and treated as riding, (C—H distances are 0.93 Å), with *U*_iso_(H) = 1.2*U*_eq_(C). The water H atoms found from Fourier difference maps were included in the refinements with restraints for O—H distances (0.829–0.833 Å) and *U*_iso_(H) = 1.5*U*_eq_(O).

### Crystal data

**Table:**

[CuEu_2_(C_6_H_4_NO_2_)_8_(H_2_O)_4_]	*F*_000_ = 1398
*M**_r_* = 1416.34	*D*_x_ = 1.801 Mg m^−^^3^
Monoclinic, *P*2_1_/*n*	Mo *K*α radiation λ = 0.71073 Å
Hall symbol: -P 2yn	Cell parameters from 6922 reflections
*a* = 9.5218 (9) Å	θ = 2.5–28.0º
*b* = 15.0371 (13) Å	µ = 2.86 mm^−^^1^
*c* = 18.2850 (16) Å	*T* = 295 (2) K
β = 93.8220 (10)º	Block, green
*V* = 2612.2 (4) Å^3^	0.40 × 0.30 × 0.27 mm
*Z* = 2	

### Data collection

**Table:**

Bruker SMART CCD area-detector diffractometer	4853 independent reflections
Radiation source: fine-focus sealed tube	4222 reflections with *I* > 2σ(*I*)
Monochromator: graphite	*R*_int_ = 0.036
*T* = 295(2) K	θ_max_ = 25.5º
φ and ω scans	θ_min_ = 2.5º
Absorption correction: multi-scan(SADABS; Sheldrick, 2003)	*h* = −10→11
*T*_min_ = 0.397, *T*_max_ = 0.517	*k* = −17→18
18387 measured reflections	*l* = −22→22

### Refinement

**Table:**

Refinement on *F*^2^	Secondary atom site location: difference Fourier map
Least-squares matrix: full	Hydrogen site location: inferred from neighbouring sites
*R*[*F*^2^ > 2σ(*F*^2^)] = 0.021	H-atom parameters constrained
*wR*(*F*^2^) = 0.049	*w* = 1/[σ^2^(*F*_o_^2^) + (0.0175*P*)^2^ + 1.449*P*] where *P* = (*F*_o_^2^ + 2*F*_c_^2^)/3
*S* = 1.04	(Δ/σ)_max_ = 0.006
4853 reflections	Δρ_max_ = 0.36 e Å^−^^3^
359 parameters	Δρ_min_ = −0.36 e Å^−^^3^
6 restraints	Extinction correction: SHELXL97 (Sheldrick, 2008), Fc^*^=kFc[1+0.001xFc^2^λ^3^/sin(2θ)]^-1/4^
Primary atom site location: structure-invariant direct methods	Extinction coefficient: 0.00683 (17)

### Special details

**Table:**

Geometry. All e.s.d.'s (except the e.s.d. in the dihedral angle between two l.s. planes)are estimated using the full covariance matrix. The cell e.s.d.'s are takeninto account individually in the estimation of e.s.d.'s in distances, anglesand torsion angles; correlations between e.s.d.'s in cell parameters are onlyused when they are defined by crystal symmetry. An approximate (isotropic)treatment of cell e.s.d.'s is used for estimating e.s.d.'s involving l.s. planes.
Refinement. Refinement of *F*^2^ against ALL reflections. The weighted *R*-factor *wR* andgoodness of fit *S* are based on *F*^2^, conventional *R*-factors *R* are basedon *F*, with *F* set to zero for negative *F*^2^. The threshold expression of*F*^2^ > σ(*F*^2^) is used only for calculating *R*-factors(gt) *etc*. and isnot relevant to the choice of reflections for refinement. *R*-factors basedon *F*^2^ are statistically about twice as large as those based on *F*, and *R*-factors based on ALL data will be even larger.

### Fractional atomic coordinates and isotropic or equivalent isotropic displacement parameters (Å^2^)

**Table:**

	*x*	*y*	*z*	*U*_iso_*/*U*_eq_	
Eu1	0.303549 (14)	1.084800 (9)	−0.000640 (7)	0.01521 (6)	
Cu1	0.0000	0.5000	0.0000	0.02112 (12)	
O1	0.1022 (2)	1.06985 (13)	0.08317 (11)	0.0256 (5)	
O2	0.2241 (2)	1.19395 (13)	0.08933 (11)	0.0319 (5)	
O3	0.4104 (2)	1.03423 (18)	0.11132 (12)	0.0440 (6)	
O4	0.6048 (3)	0.95248 (17)	0.11231 (12)	0.0437 (6)	
O5	0.2714 (2)	0.92881 (14)	−0.00398 (13)	0.0378 (6)	
O6	0.4704 (2)	0.85317 (15)	−0.00984 (14)	0.0454 (6)	
O7	−0.1420 (2)	0.55366 (14)	−0.07136 (11)	0.0276 (5)	
O8	−0.3139 (2)	0.62128 (16)	−0.01438 (11)	0.0370 (6)	
N1	−0.0769 (4)	1.2279 (2)	0.30315 (16)	0.0494 (8)	
N2	0.5926 (3)	1.0505 (3)	0.37273 (15)	0.0521 (9)	
N3	0.1135 (2)	0.61288 (15)	0.00239 (12)	0.0193 (5)	
N4	−0.4825 (3)	0.6230 (2)	−0.28160 (15)	0.0448 (8)	
C1	−0.1334 (4)	1.1683 (3)	0.2564 (2)	0.0579 (12)	
H1	−0.2213	1.1456	0.2656	0.069*	
C2	−0.0711 (4)	1.1377 (3)	0.1950 (2)	0.0463 (10)	
H2	−0.1151	1.0949	0.1648	0.056*	
C3	0.0576 (3)	1.1721 (2)	0.17983 (15)	0.0259 (7)	
C4	0.1180 (4)	1.2343 (2)	0.22750 (18)	0.0420 (9)	
H4	0.2050	1.2589	0.2189	0.050*	
C5	0.0485 (4)	1.2599 (3)	0.2882 (2)	0.0507 (10)	
H5	0.0914	1.3015	0.3201	0.061*	
C6	0.1329 (3)	1.1432 (2)	0.11331 (15)	0.0226 (6)	
C7	0.5089 (4)	1.1027 (3)	0.33001 (19)	0.0462 (10)	
H7	0.4656	1.1504	0.3518	0.055*	
C8	0.4824 (4)	1.0906 (2)	0.25533 (17)	0.0314 (7)	
H8	0.4216	1.1283	0.2282	0.038*	
C9	0.5483 (3)	1.0211 (2)	0.22209 (15)	0.0229 (6)	
C10	0.6410 (4)	0.9697 (3)	0.26468 (17)	0.0406 (9)	
H10	0.6913	0.9242	0.2439	0.049*	
C11	0.6578 (4)	0.9867 (3)	0.33875 (19)	0.0573 (12)	
H11	0.7197	0.9507	0.3669	0.069*	
C12	0.5193 (3)	1.0016 (2)	0.14180 (15)	0.0252 (7)	
C13	0.0470 (3)	0.69169 (19)	0.00148 (15)	0.0233 (6)	
H13	−0.0508	0.6921	0.0004	0.028*	
C14	0.1172 (3)	0.77197 (19)	0.00207 (15)	0.0231 (6)	
H14	0.0676	0.8252	0.0027	0.028*	
C15	0.2621 (3)	0.77222 (19)	0.00173 (14)	0.0207 (6)	
C16	0.3327 (3)	0.69096 (19)	0.00510 (15)	0.0241 (7)	
H16	0.4305	0.6889	0.0072	0.029*	
C17	0.2545 (3)	0.6135 (2)	0.00524 (15)	0.0229 (6)	
H17	0.3018	0.5594	0.0074	0.027*	
C18	0.3420 (3)	0.8586 (2)	−0.00392 (16)	0.0254 (7)	
C19	−0.5216 (3)	0.6665 (2)	−0.22291 (19)	0.0408 (9)	
H19	−0.5995	0.7038	−0.2288	0.049*	
C20	−0.4541 (3)	0.6600 (2)	−0.15378 (17)	0.0320 (7)	
H20	−0.4856	0.6926	−0.1148	0.038*	
C21	−0.3387 (3)	0.6041 (2)	−0.14357 (16)	0.0258 (7)	
C22	−0.2961 (4)	0.5588 (2)	−0.20395 (18)	0.0420 (9)	
H22	−0.2191	0.5207	−0.1994	0.050*	
C23	−0.3693 (4)	0.5708 (3)	−0.2715 (2)	0.0532 (11)	
H23	−0.3379	0.5410	−0.3118	0.064*	
C24	−0.2594 (3)	0.59323 (18)	−0.06933 (16)	0.0239 (7)	
O9	0.0897 (2)	1.07055 (13)	−0.07832 (11)	0.0284 (5)	
H1W	0.0757	1.0919	−0.1202	0.043*	
H2W	0.0301	1.0314	−0.0715	0.043*	
O10	0.2777 (2)	1.22483 (13)	−0.06923 (11)	0.0299 (5)	
H3W	0.3208	1.2307	−0.1068	0.045*	
H4W	0.2738	1.2729	−0.0473	0.045*	

### Atomic displacement parameters (Å^2^)

**Table:**

	*U*^11^	*U*^22^	*U*^33^	*U*^12^	*U*^13^	*U*^23^
Eu1	0.01495 (9)	0.01529 (9)	0.01549 (8)	−0.00210 (6)	0.00169 (5)	−0.00087 (5)
Cu1	0.0205 (3)	0.0153 (3)	0.0264 (3)	−0.0064 (2)	−0.0071 (2)	0.0056 (2)
O1	0.0249 (12)	0.0272 (12)	0.0251 (11)	−0.0042 (9)	0.0061 (9)	−0.0054 (9)
O2	0.0394 (13)	0.0233 (12)	0.0351 (12)	−0.0072 (10)	0.0194 (10)	−0.0044 (9)
O3	0.0394 (15)	0.0656 (18)	0.0255 (12)	0.0092 (13)	−0.0078 (11)	0.0041 (12)
O4	0.0550 (16)	0.0506 (15)	0.0274 (12)	0.0129 (13)	0.0161 (11)	−0.0074 (11)
O5	0.0373 (14)	0.0178 (12)	0.0576 (16)	−0.0060 (10)	−0.0016 (12)	0.0012 (10)
O6	0.0214 (13)	0.0354 (14)	0.0796 (19)	−0.0127 (11)	0.0041 (12)	0.0022 (13)
O7	0.0268 (12)	0.0267 (12)	0.0281 (11)	−0.0024 (10)	−0.0083 (9)	0.0054 (9)
O8	0.0475 (15)	0.0358 (14)	0.0272 (12)	0.0008 (11)	−0.0022 (11)	−0.0020 (10)
N1	0.064 (2)	0.0436 (19)	0.0442 (18)	−0.0060 (17)	0.0313 (16)	−0.0110 (15)
N2	0.046 (2)	0.088 (3)	0.0216 (15)	−0.0102 (19)	−0.0030 (14)	−0.0089 (16)
N3	0.0209 (13)	0.0159 (12)	0.0208 (12)	−0.0044 (10)	−0.0011 (10)	0.0023 (9)
N4	0.049 (2)	0.0461 (19)	0.0368 (17)	0.0086 (16)	−0.0195 (14)	−0.0070 (14)
C1	0.049 (2)	0.062 (3)	0.067 (3)	−0.017 (2)	0.040 (2)	−0.023 (2)
C2	0.037 (2)	0.050 (2)	0.054 (2)	−0.0138 (18)	0.0207 (18)	−0.0225 (18)
C3	0.0276 (17)	0.0260 (17)	0.0250 (16)	0.0029 (14)	0.0077 (13)	−0.0016 (13)
C4	0.045 (2)	0.043 (2)	0.041 (2)	−0.0128 (18)	0.0190 (17)	−0.0108 (16)
C5	0.065 (3)	0.045 (2)	0.044 (2)	−0.013 (2)	0.0199 (19)	−0.0180 (18)
C6	0.0195 (16)	0.0263 (17)	0.0219 (15)	0.0037 (13)	0.0021 (12)	0.0027 (12)
C7	0.057 (3)	0.050 (2)	0.034 (2)	−0.007 (2)	0.0143 (18)	−0.0162 (17)
C8	0.0341 (19)	0.0342 (19)	0.0267 (16)	0.0047 (15)	0.0072 (14)	−0.0006 (14)
C9	0.0208 (16)	0.0294 (17)	0.0184 (14)	−0.0025 (13)	0.0018 (12)	0.0014 (12)
C10	0.040 (2)	0.054 (2)	0.0282 (18)	0.0150 (18)	0.0034 (15)	0.0054 (16)
C11	0.046 (2)	0.100 (4)	0.0249 (19)	0.018 (2)	−0.0035 (17)	0.011 (2)
C12	0.0283 (18)	0.0276 (17)	0.0198 (15)	−0.0068 (14)	0.0032 (13)	0.0029 (13)
C13	0.0177 (15)	0.0229 (16)	0.0289 (16)	−0.0017 (12)	−0.0013 (12)	0.0014 (12)
C14	0.0211 (16)	0.0169 (15)	0.0313 (16)	0.0008 (12)	0.0003 (12)	0.0002 (12)
C15	0.0217 (16)	0.0209 (16)	0.0190 (14)	−0.0076 (12)	−0.0011 (12)	−0.0006 (11)
C16	0.0168 (16)	0.0236 (17)	0.0319 (16)	−0.0036 (12)	0.0015 (13)	0.0012 (13)
C17	0.0221 (16)	0.0184 (15)	0.0278 (16)	0.0002 (13)	−0.0006 (12)	0.0017 (12)
C18	0.0270 (18)	0.0219 (17)	0.0268 (16)	−0.0085 (13)	−0.0021 (13)	0.0001 (12)
C19	0.0283 (19)	0.045 (2)	0.047 (2)	0.0097 (17)	−0.0104 (16)	0.0023 (17)
C20	0.0272 (18)	0.037 (2)	0.0319 (17)	0.0040 (15)	−0.0007 (14)	−0.0001 (14)
C21	0.0246 (17)	0.0228 (17)	0.0291 (16)	−0.0030 (13)	−0.0060 (13)	0.0019 (12)
C22	0.046 (2)	0.042 (2)	0.0360 (19)	0.0178 (18)	−0.0124 (17)	−0.0118 (16)
C23	0.062 (3)	0.057 (3)	0.038 (2)	0.022 (2)	−0.0196 (19)	−0.0229 (18)
C24	0.0262 (17)	0.0156 (15)	0.0289 (16)	−0.0055 (13)	−0.0055 (13)	0.0038 (12)
O9	0.0247 (12)	0.0343 (13)	0.0250 (11)	−0.0101 (10)	−0.0081 (9)	0.0053 (9)
O10	0.0414 (13)	0.0221 (11)	0.0277 (11)	0.0021 (10)	0.0129 (10)	0.0045 (9)

### Geometric parameters (Å, °)

**Table:**

Eu1—O4^i^	2.341 (2)	C3—C6	1.516 (4)
Eu1—O6^i^	2.342 (2)	C4—C5	1.385 (5)
Eu1—O3	2.351 (2)	C4—H4	0.9300
Eu1—O5	2.366 (2)	C5—H5	0.9300
Eu1—O9	2.4133 (19)	C7—C8	1.384 (5)
Eu1—O10	2.4548 (19)	C7—H7	0.9300
Eu1—O2	2.477 (2)	C8—C9	1.381 (4)
Eu1—O1	2.5428 (19)	C8—H8	0.9300
Eu1—C6	2.864 (3)	C9—C10	1.376 (4)
Cu1—O7^ii^	1.9867 (19)	C9—C12	1.504 (4)
Cu1—O7	1.9867 (19)	C10—C11	1.377 (5)
Cu1—N3	2.011 (2)	C10—H10	0.9300
Cu1—N3^ii^	2.011 (2)	C11—H11	0.9300
O1—C6	1.259 (3)	C13—C14	1.380 (4)
O2—C6	1.256 (3)	C13—H13	0.9300
O3—C12	1.245 (4)	C14—C15	1.380 (4)
O4—C12	1.248 (4)	C14—H14	0.9300
O4—Eu1^i^	2.341 (2)	C15—C16	1.394 (4)
O5—C18	1.251 (4)	C15—C18	1.513 (4)
O6—C18	1.237 (4)	C16—C17	1.383 (4)
O6—Eu1^i^	2.342 (2)	C16—H16	0.9300
O7—C24	1.269 (4)	C17—H17	0.9300
O8—C24	1.235 (4)	C19—C20	1.383 (4)
N1—C1	1.326 (5)	C19—H19	0.9300
N1—C5	1.333 (5)	C20—C21	1.386 (4)
N2—C11	1.320 (5)	C20—H20	0.9300
N2—C7	1.334 (5)	C21—C22	1.381 (4)
N3—C17	1.340 (4)	C21—C24	1.517 (4)
N3—C13	1.343 (4)	C22—C23	1.389 (5)
N4—C19	1.331 (4)	C22—H22	0.9300
N4—C23	1.336 (5)	C23—H23	0.9300
C1—C2	1.382 (5)	O9—H1W	0.8334
C1—H1	0.9300	O9—H2W	0.8326
C2—C3	1.376 (4)	O10—H3W	0.8291
C2—H2	0.9300	O10—H4W	0.8292
C3—C4	1.378 (4)		
			
O4^i^—Eu1—O6^i^	76.87 (9)	N1—C5—H5	118.5
O4^i^—Eu1—O3	121.14 (8)	C4—C5—H5	118.5
O6^i^—Eu1—O3	73.47 (9)	O2—C6—O1	121.7 (3)
O4^i^—Eu1—O5	78.18 (9)	O2—C6—C3	118.8 (3)
O6^i^—Eu1—O5	120.90 (8)	O1—C6—C3	119.6 (3)
O3—Eu1—O5	75.49 (9)	O2—C6—Eu1	59.57 (14)
O4^i^—Eu1—O9	79.30 (8)	O1—C6—Eu1	62.56 (14)
O6^i^—Eu1—O9	144.92 (8)	C3—C6—Eu1	173.2 (2)
O3—Eu1—O9	141.54 (8)	N2—C7—C8	124.3 (3)
O5—Eu1—O9	78.12 (7)	N2—C7—H7	117.9
O4^i^—Eu1—O10	77.90 (8)	C8—C7—H7	117.9
O6^i^—Eu1—O10	76.15 (8)	C7—C8—C9	118.4 (3)
O3—Eu1—O10	138.18 (8)	C7—C8—H8	120.8
O5—Eu1—O10	145.98 (7)	C9—C8—H8	120.8
O9—Eu1—O10	73.94 (7)	C10—C9—C8	118.0 (3)
O4^i^—Eu1—O2	152.34 (8)	C10—C9—C12	120.8 (3)
O6^i^—Eu1—O2	90.07 (8)	C8—C9—C12	121.2 (3)
O3—Eu1—O2	76.68 (8)	C9—C10—C11	118.7 (3)
O5—Eu1—O2	129.00 (7)	C9—C10—H10	120.6
O9—Eu1—O2	99.60 (7)	C11—C10—H10	120.6
O10—Eu1—O2	75.30 (7)	N2—C11—C10	124.8 (4)
O4^i^—Eu1—O1	147.58 (8)	N2—C11—H11	117.6
O6^i^—Eu1—O1	135.36 (8)	C10—C11—H11	117.6
O3—Eu1—O1	75.31 (7)	O3—C12—O4	126.0 (3)
O5—Eu1—O1	80.01 (7)	O3—C12—C9	117.0 (3)
O9—Eu1—O1	72.93 (7)	O4—C12—C9	117.0 (3)
O10—Eu1—O1	109.25 (7)	N3—C13—C14	122.9 (3)
O2—Eu1—O1	51.88 (6)	N3—C13—H13	118.5
O4^i^—Eu1—C6	165.98 (8)	C14—C13—H13	118.5
O6^i^—Eu1—C6	112.40 (9)	C15—C14—C13	119.1 (3)
O3—Eu1—C6	72.50 (8)	C15—C14—H14	120.4
O5—Eu1—C6	104.14 (8)	C13—C14—H14	120.4
O9—Eu1—C6	87.60 (8)	C14—C15—C16	118.5 (3)
O10—Eu1—C6	93.78 (7)	C14—C15—C18	120.7 (3)
O2—Eu1—C6	25.93 (7)	C16—C15—C18	120.8 (3)
O1—Eu1—C6	26.07 (7)	C17—C16—C15	118.7 (3)
O7^ii^—Cu1—O7	180.0	C17—C16—H16	120.7
O7^ii^—Cu1—N3	89.26 (9)	C15—C16—H16	120.7
O7—Cu1—N3	90.74 (9)	N3—C17—C16	123.0 (3)
O7^ii^—Cu1—N3^ii^	90.74 (9)	N3—C17—H17	118.5
O7—Cu1—N3^ii^	89.26 (9)	C16—C17—H17	118.5
N3—Cu1—N3^ii^	180.0	O6—C18—O5	126.2 (3)
C6—O1—Eu1	91.37 (16)	O6—C18—C15	116.9 (3)
C6—O2—Eu1	94.50 (17)	O5—C18—C15	116.8 (3)
C12—O3—Eu1	144.7 (2)	N4—C19—C20	124.4 (3)
C12—O4—Eu1^i^	144.1 (2)	N4—C19—H19	117.8
C18—O5—Eu1	140.1 (2)	C20—C19—H19	117.8
C18—O6—Eu1^i^	151.0 (2)	C19—C20—C21	118.8 (3)
C24—O7—Cu1	137.19 (19)	C19—C20—H20	120.6
C1—N1—C5	116.3 (3)	C21—C20—H20	120.6
C11—N2—C7	115.7 (3)	C22—C21—C20	117.6 (3)
C17—N3—C13	117.7 (2)	C22—C21—C24	120.5 (3)
C17—N3—Cu1	122.85 (19)	C20—C21—C24	121.9 (3)
C13—N3—Cu1	119.46 (19)	C21—C22—C23	119.3 (3)
C19—N4—C23	116.3 (3)	C21—C22—H22	120.3
N1—C1—C2	124.8 (3)	C23—C22—H22	120.3
N1—C1—H1	117.6	N4—C23—C22	123.5 (3)
C2—C1—H1	117.6	N4—C23—H23	118.2
C3—C2—C1	118.3 (3)	C22—C23—H23	118.2
C3—C2—H2	120.8	O8—C24—O7	127.0 (3)
C1—C2—H2	120.8	O8—C24—C21	118.7 (3)
C2—C3—C4	117.9 (3)	O7—C24—C21	114.3 (3)
C2—C3—C6	122.0 (3)	Eu1—O9—H1W	125.7
C4—C3—C6	120.1 (3)	Eu1—O9—H2W	122.1
C3—C4—C5	119.6 (3)	H1W—O9—H2W	110.0
C3—C4—H4	120.2	Eu1—O10—H3W	118.5
C5—C4—H4	120.2	Eu1—O10—H4W	120.4
N1—C5—C4	123.1 (3)	H3W—O10—H4W	110.4
			
O4^i^—Eu1—O1—C6	−154.11 (17)	O10—Eu1—C6—O2	−44.17 (18)
O6^i^—Eu1—O1—C6	33.3 (2)	O1—Eu1—C6—O2	−172.2 (3)
O3—Eu1—O1—C6	80.12 (17)	O4^i^—Eu1—C6—O1	75.1 (4)
O5—Eu1—O1—C6	157.60 (17)	O6^i^—Eu1—C6—O1	−155.36 (16)
O9—Eu1—O1—C6	−121.87 (17)	O3—Eu1—C6—O1	−92.26 (17)
O10—Eu1—O1—C6	−56.31 (17)	O5—Eu1—C6—O1	−22.77 (18)
O2—Eu1—O1—C6	−4.30 (16)	O9—Eu1—C6—O1	54.35 (16)
O4^i^—Eu1—O2—C6	148.82 (19)	O10—Eu1—C6—O1	128.07 (16)
O6^i^—Eu1—O2—C6	−150.30 (18)	O2—Eu1—C6—O1	172.2 (3)
O3—Eu1—O2—C6	−77.32 (18)	C11—N2—C7—C8	3.6 (6)
O5—Eu1—O2—C6	−18.9 (2)	N2—C7—C8—C9	−1.3 (6)
O9—Eu1—O2—C6	63.58 (18)	C7—C8—C9—C10	−2.4 (5)
O10—Eu1—O2—C6	134.05 (18)	C7—C8—C9—C12	177.2 (3)
O1—Eu1—O2—C6	4.33 (16)	C8—C9—C10—C11	3.5 (5)
O4^i^—Eu1—O3—C12	16.4 (4)	C12—C9—C10—C11	−176.2 (3)
O6^i^—Eu1—O3—C12	−46.5 (4)	C7—N2—C11—C10	−2.4 (6)
O5—Eu1—O3—C12	82.6 (4)	C9—C10—C11—N2	−1.1 (7)
O9—Eu1—O3—C12	130.7 (4)	Eu1—O3—C12—O4	−29.2 (6)
O10—Eu1—O3—C12	−91.6 (4)	Eu1—O3—C12—C9	152.7 (3)
O2—Eu1—O3—C12	−140.6 (4)	Eu1^i^—O4—C12—O3	26.5 (6)
O1—Eu1—O3—C12	165.8 (4)	Eu1^i^—O4—C12—C9	−155.4 (3)
C6—Eu1—O3—C12	−167.2 (4)	C10—C9—C12—O3	161.7 (3)
O4^i^—Eu1—O5—C18	64.0 (3)	C8—C9—C12—O3	−17.9 (4)
O6^i^—Eu1—O5—C18	−2.7 (4)	C10—C9—C12—O4	−16.6 (4)
O3—Eu1—O5—C18	−62.9 (3)	C8—C9—C12—O4	163.8 (3)
O9—Eu1—O5—C18	145.3 (3)	C17—N3—C13—C14	1.1 (4)
O10—Eu1—O5—C18	110.1 (3)	Cu1—N3—C13—C14	−179.2 (2)
O2—Eu1—O5—C18	−121.8 (3)	N3—C13—C14—C15	1.7 (4)
O1—Eu1—O5—C18	−140.2 (3)	C13—C14—C15—C16	−3.7 (4)
C6—Eu1—O5—C18	−130.2 (3)	C13—C14—C15—C18	175.0 (3)
N3—Cu1—O7—C24	−95.7 (3)	C14—C15—C16—C17	2.9 (4)
N3^ii^—Cu1—O7—C24	84.3 (3)	C18—C15—C16—C17	−175.7 (3)
O7^ii^—Cu1—N3—C17	39.1 (2)	C13—N3—C17—C16	−1.9 (4)
O7—Cu1—N3—C17	−140.9 (2)	Cu1—N3—C17—C16	178.4 (2)
O7^ii^—Cu1—N3—C13	−140.6 (2)	C15—C16—C17—N3	−0.1 (4)
O7—Cu1—N3—C13	39.4 (2)	Eu1^i^—O6—C18—O5	22.8 (7)
C5—N1—C1—C2	0.6 (7)	Eu1^i^—O6—C18—C15	−159.1 (3)
N1—C1—C2—C3	−1.4 (7)	Eu1—O5—C18—O6	−8.6 (5)
C1—C2—C3—C4	1.0 (6)	Eu1—O5—C18—C15	173.3 (2)
C1—C2—C3—C6	−179.3 (3)	C14—C15—C18—O6	−174.3 (3)
C2—C3—C4—C5	−0.1 (5)	C16—C15—C18—O6	4.3 (4)
C6—C3—C4—C5	−179.8 (3)	C14—C15—C18—O5	3.9 (4)
C1—N1—C5—C4	0.4 (6)	C16—C15—C18—O5	−177.5 (3)
C3—C4—C5—N1	−0.7 (6)	C23—N4—C19—C20	−0.7 (6)
Eu1—O2—C6—O1	−8.1 (3)	N4—C19—C20—C21	−0.7 (5)
Eu1—O2—C6—C3	172.4 (2)	C19—C20—C21—C22	1.1 (5)
Eu1—O1—C6—O2	7.9 (3)	C19—C20—C21—C24	−179.8 (3)
Eu1—O1—C6—C3	−172.7 (2)	C20—C21—C22—C23	0.0 (5)
C2—C3—C6—O2	158.1 (3)	C24—C21—C22—C23	−179.1 (3)
C4—C3—C6—O2	−22.2 (4)	C19—N4—C23—C22	1.9 (6)
C2—C3—C6—O1	−21.4 (5)	C21—C22—C23—N4	−1.5 (7)
C4—C3—C6—O1	158.3 (3)	Cu1—O7—C24—O8	10.8 (5)
O4^i^—Eu1—C6—O2	−97.1 (4)	Cu1—O7—C24—C21	−168.0 (2)
O6^i^—Eu1—C6—O2	32.40 (19)	C22—C21—C24—O8	−167.1 (3)
O3—Eu1—C6—O2	95.50 (18)	C20—C21—C24—O8	13.9 (4)
O5—Eu1—C6—O2	164.99 (17)	C22—C21—C24—O7	11.8 (4)
O9—Eu1—C6—O2	−117.89 (18)	C20—C21—C24—O7	−167.3 (3)

Symmetry codes: (i) −*x*+1, −*y*+2, −*z*; (ii) −*x*, −*y*+1, −*z*.

### Hydrogen-bond geometry (Å, °)

**Table:**

*D*—H···*A*	*D*—H	H···*A*	*D*···*A*	*D*—H···*A*
O10—H4W···O8^iii^	0.83	1.97	2.782 (3)	165
O9—H2W···O1^iii^	0.83	1.98	2.790 (3)	164
O10—H3W···N1^iv^	0.83	2.06	2.881 (3)	169
O9—H1W···N4^v^	0.83	2.00	2.807 (3)	161

Symmetry codes: (iii) −*x*, −*y*+2, −*z*; (iv) *x*+1/2, −*y*+5/2, *z*−1/2; (v) −*x*−1/2, *y*+1/2, −*z*−1/2.
